# Vancomycin-resistant *Staphylococcus aureus* isolated from camel meat and slaughterhouse workers in Egypt

**DOI:** 10.1186/s13756-019-0585-4

**Published:** 2019-08-05

**Authors:** Khaled Al-Amery, Mahmoud Elhariri, Alaa Elsayed, Gihan El-Moghazy, Rehab Elhelw, Heba El-Mahallawy, Mohamed El Hariri, Dalia Hamza

**Affiliations:** 1 Department of Microbiology, Faculty of Veterinary Medicine, Cairo University, PO Box 12211, Cairo, Egypt; 2 Department of Food Safety and Biotechnology, Regional Center for Food and Feed, Agricultural Research Center, Giza, Egypt; 3 Department of Animal Hygiene, Zoonoses and Animal Behaviour and Management, Faculty of Veterinary Medicine, Suez Canal University, Ismailia, 41522 Egypt; 4 Department of Dermatology, Venereology and Andrology, Faculty of Medicine, Alazhar University, Cairo, Egypt; 5 Department of Zoonoses, Faculty of Veterinary Medicine, Cairo University, PO Box 12211, Giza, Cairo, Egypt

**Keywords:** Dromedary camels, Human, *S. aureus*, VRSA, Abattoir, Egypt

## Abstract

**Background:**

The emergence of vancomycin-resistant *Staphylococcus aureus* (VRSA) represents a challenge for the treatment of staphylococcal infections in both human and animals worldwide. Although VRSA has been detected in several animal species worldwide, data on the bacterial prevalence in dromedary camels and workers in camel slaughterhouses are scarce.

**Methods:**

We investigated meat samples from 200 dromedary camel carcasses from three different abattoirs that were being prepared to be sent to the markets. Twenty hand swabs were voluntarily collected from the workers in the same abattoirs. Isolation and identification of the bacterial specimens from the samples were performed using conventional cultural techniques and biochemical identification and were confirmed by PCR amplification of the *nuc* gene. Antimicrobial susceptibility against nine antimicrobial agents commonly used in human and camels was tested using the disc diffusion method, and genetic analysis was performed by evaluating the *mecA* gene in phenotypically oxacillin (OXA)- and cefoxitin (FOX)-resistant isolates. The resistance of *S. aureus* to vancomycin (VAN) was tested by broth microdilution and confirmed by PCR targeting the *vanA* and *vanB* genes. The *vanA* and *vanB* genes were sequenced.

**Result:**

*S. aureus* was detected in both camel meat (29/200, 14.5%) and in abattoir workers (11/20, 55%). Of the collected samples, 27% (8/29, camel) and 54% (6/11, human) were identified as VRSA.

All VRSA isolates carried both the *vanA* and *vanB* genes. Additionally, all VRSA isolates were also classified as methicillin-resistant *S. aureus* (MRSA). The *vanA* amplicons of the isolates from human and camel meat were homologous and clustered with a Chinese reference isolate sequence.

**Conclusion:**

This study demonstrated that VRSA is present in camel abattoirs in Egypt. Zoonotic transmission between animals and human is probable and reflects both a public health threat and a food safety concern.

## Background

*Staphylococcus aureus* (*S. aureus*) is one of the most common microorganisms that colonize the nasal cavity and/or the external body surfaces of human and various animal species. *S. aureus* may be present either as commensal bacteria or pathogenic bacteria, which can cause multiple infectious diseases [1]. Since the first report of a methicillin-resistant *S. aureus* (MRSA) strain in 1961 from a human patient, attention has been paid to its public health significance, leaving vancomycin (VAN) as the antibiotic of choice for the treatment of many infections [2]. However, in July 2002, the situation changed when the Centers for Disease Control and Prevention (CDC) in the USA documented the first sample of *S. aureus* that was resistant to both VAN and methicillin [3].

In the Middle East, the dromedary camel (*Camelus dromedarius*, one-humped camel) is an important livestock species adapted to hot and dry environments. In Egypt, camels are frequently slaughtered, and their meat is consumed by human year-round.

Camels were formerly thought not to be affected by most of the diseases commonly impacting livestock; however, recent data have confirmed their susceptibility to a high number of pathogens, and camels are currently believed to act as a carrier or reservoir for the transmission of several transboundary animal diseases and zoonoses [4].

Epidemiological studies on resistant *S. aureus* in camels usually focus on the bacterial prevalence in milk [5–7]; few studies have discussed anthropozoonotic transmission vs. zooanthroponotic transmission due to contact with camels by slaughterhouse employees or camel breeders.

In Egypt, no data are available about the distribution, colonization, and transmission of resistant *S. aureus* in camels and their human contacts. This study was carried out to determine the occurrence of VRSA among dromedary camels and slaughterhouse workers and to study the probable zoonotic risk.

## Materials and methods

### Sample collection

#### Camel meat samples

Two hundred meat samples were collected from 200 camel carcasses (one sample from each animal) after slaughter from three different abattoirs in the greater Cairo area (GCA); samples were collected throughout 2017.

#### Human hand swabs

Hand swabs were collected from 20 adult male slaughterhouse workers. All workers were informed about the nature of the experiment. Sample collection was performed after handling meat for no less than 1 hour. All the workers were clinically free from any bacterial skin infections at the time of examination. Workers were asked not to wash their hands before sampling.

The palm surfaces of both hands were swabbed with cotton tipped swabs moistened with sterile saline. The entire palm surface was swabbed perpendicularly. We avoided obtaining samples from interdigital areas. Sterile gloves were used during sampling to minimize sample cross-contamination. Sample blanks consisted of swabs that had been moistened and placed directly in sterile 15-ml polypropylene tubes. Following collection, all samples were transported on ice to the Faculty of Veterinary Medicine, Cairo University, where they were processed for *Staphylococcus* spp. isolation.

### Isolation and identification of *S. aureus*

One gram of meat samples from the animals and the hand swabs from the workers were placed into 9 ml of brain heart infusion broth (Oxoid, Hampshire, UK) and incubated at 37 °C for 24 h. Two loopfuls from each broth sample were plated on mannitol salt agar (Oxoid, Hampshire, UK) and 5% sheep blood agar (Oxoid Ltd., Hampshire, UK) and incubated aerobically at 37 °C for 24 h.

The typical *Staphylococcus* spp. colonies were further examined by gram staining and traditional biochemical methods according to Quinn [8] and confirmed as *S. aureus* by both the latex agglutination test using a Staphytect Plus kit (Oxoid, UK); *nuc* gene detection was performed according to **Louie et al., 2002** [9].

At least two colonies from each positive plate were maintained on brain heart infusion broth for further testing and PCR analysis.

### Antimicrobial susceptibility test

#### Disc agar diffusion test

The Kirby-Bauer disc diffusion technique was performed to determine the antibiotic resistance profile of the isolates. After overnight incubation on Mueller-Hinton agar at 37 °C (Oxoid Ltd., Hampshire, UK), the inhibition zones were measured, and the interpretation was carried out according to the Clinical and Laboratory Standards Institute (CLSI) guidelines [10]. *S. aureus* isolates were tested against nine different antibiotics with the following corresponding concentrations: chloramphenicol (CHL) (30 μg/disc), clindamycin (CLI) (2 μg/disc), erythromycin (ERY) (15 μg/disc), novobiocin (NV) (30 μg/disc), ofloxacin (OFX) (5 μg/disc), cefoxitin (FOX) (30 μg/disc), oxacillin (OXA) (1 μg/disc), trimethoprim-sulfamethoxazole (SXT) (23.75 μg/disc) and VAN (30 μg/disc). The discs were purchased from Oxoid Ltd. (Hampshire, UK).

#### Determination of minimum inhibitory concentration

The minimum inhibitory concentration (MIC) values of VAN were determined by a broth microdilution method using cation-adjusted Mueller-Hinton broth (Oxoid Ltd., Hampshire, UK) and VAN standard antibiotic (Sigma Aldrich). The procedure and interpretation of the results were performed according to the CLSI guidelines [10]. The laboratory breakpoints were as follows: vancomycin-susceptible *S. aureus* (VSSA) = vancomycin MIC < 2 μg/ml; and VRSA = vancomycin MIC > 16 μg/ml.

### DNA extraction

All *S. aureus* isolates were grown on mannitol salt agar at 37 °C overnight. A single bacterial colony from each plate was picked and suspended in 200 μl deionized distilled water. Genomic DNA was extracted using the QIAamp Mini DNA Extraction Kit (Qiagen, Hilden, Germany).

#### Molecular confirmation of *S. aureus,* MRSA and VRSA isolates

(i) Molecular confirmation was performed by amplification of the *S. aureus*-specific *nuc* gene to identify positive *S. aureus* isolates [9].
(ii) PCR identification of the *mecA* gene was performed in phenotypically FOX- and OXA-resistant isolates (25 isolates).
(iii) PCR amplification of *vanA* and *vanB* genes encoding VAN resistance was conducted in phenotypically VAN-resistant isolates (14 isolates).

*Staphylococcus aureus* ATCC 29213 and *Enterococcus faecalis* ATCC 29212 strains were used as VAN-susceptible controls [11]. VAN-resistant *Enterococcus faecium* ATCC 51559 was used as a *vanA*-positive control strain, and *E. faecalis* ATCC 51299 was used as a *vanB*-positive control strain.

PCR amplification was performed using 3 μl of the extracted bacterial DNA, 25 μl of 2X DreamTaq DNA PCR Master Mix (Thermo Scientific, Waltham, USA), and 0.5 μl of each primer at a concentration of 20 pmol; nuclease-free water was added up to 50 μl. The primer pairs and cycling conditions used in the PCRs are summarized in Table 1.

**Table 1:** List of primer pairs and cycling conditions for the *nuc*, *mecA, vanA* and *vanB* genes used in this study

Target gene	*nuc*	*mecA*	*vanA*	*vanB*
Primer pairs	5′-GCGATTGATGGTGATACGGTT-3′ 5′-AGCCAAGCCTTGACGAACTAAAGC-3’	5’-AGAAGATGGTATGTGGAAGTTAG--3′ 5′-ATGTATGTGCGATTGTATTGC-3’	5’- GGCAAGTCAGGTGAAGATG-3′ 5’ ATCAAGCGGTCAATCAGTTC-3’	5’ GTG ACA AAC CGG AGG CGA GGA 3′ 5′ CCG CCA TCC TCC TGC AAA AAA-3’
PCR product (bp)	270	583	713	430
Cycling conditions	• Initial denaturation at 94 °C for 5 min. (35 cycles): • Denaturation at 94 °C for 30 s. • Annealing at 55 °C for 30 s. • Polymerization at 72 °C for 1 min. • Final extension step at 72 °C and 10 min. Louie et al., 2002 [9].	• Initial denaturation at 94 °C for 5 min. (40 cycles): • Denaturation at 94 °C for 30 s. • Annealing at 57 °C for 45 s. • Polymerization at 72 °C for 30 s. • Final extension step at 72 °C and 5 min. Azimian et al., 2012 [12].	• Initial denaturation at 94 °C for 5 min. (40 cycles): • Denaturation at 94 °C for I min. • Annealing at 55 °C for 1 min. • Polymerization at 72 °C for 2 min. • Final extension step at 72 °C and 5 min. Azimian et al., 2012 [12].	• Initial denaturation at 94 °C for 10 min. (30 cycles): • Denaturation step at 94 °C and 30 s. • Annealing step at 50 °C and a 45 s. • Polymerization at 72 °C for 30 s. • Final extension step at 72 °C and 10 min. Saadat et al., 2014 [11].

Fifteen microlitres of the amplification products were identified by electrophoresis in a 1.5% agarose gel (Sigma, Darmstadt, Germany) stained with 1 μg/ml of ethidium bromide (Sigma, Darmstadt, Germany) in 1x TAE buffer for 30 min before being visualized under UV light and photographed.

### Sequencing and nucleotide sequence analysis

The amplification products of four VRSA isolates (two camels and two human VRSA-positive isolates) were sequenced at Promega Lab Technology (Madison, USA) using the forward and reverse primers of the *vanA* and *vanB* genes after being purified from the gel using a QIAquick gel extraction kit (Qiagen, Hilden, Germany) according to the manufacturer’s instructions. The sequence was deposited in the GenBank database under the accession numbers for the *vanA* gene (MH744353 and MH744354 for the camel meat isolates and MH744355 and MH744356 for the human isolates). The accession numbers for the *vanB* gene are MK087830 and MK087831 for the camel meat isolates and MK087832 and MK095504 for the human isolates.

The nucleotide sequences of the *vanA* isolates were compared with the sequences available in the public domains using the National Center for Biotechnology Information (NCBI) Basic Local Alignment Search Tool (BLAST) server. Sequences were downloaded and imported into BIOEDIT version 7.0.1.4 for multiple alignments according to their deduced amino acid sequences using the CLUSTALW program of BIOEDIT.

Nucleotide sequence analysis was performed using MEGA version 7 with the neighbour-joining approach. Bootstrap analysis was performed with 1000 resamplings.

### Statistical analysis

PASW statistics by SPSS 18.0 (SPSS Inc., Chicago, IL, USA) was used to analyse the data. Chi-square and Fisher’s exact tests were used to compare carriage rates between different abattoirs and hosts and sensitivity to different antibiotics. Differences were considered statistically significant if the *P* value was < 0.05.

### Ethics statement

Protocols for the collection of samples were conducted according to the guidelines of the Institutional Animal Care and Use Committee (IACUC) of the Faculty of Veterinary Medicine, Cairo University, Egypt (VetCU05192019041).

Oral consent was obtained from each abattoir worker who participated in the study after they were educated on the use of the hand swab samples.

## Results

**Fig. 1:**
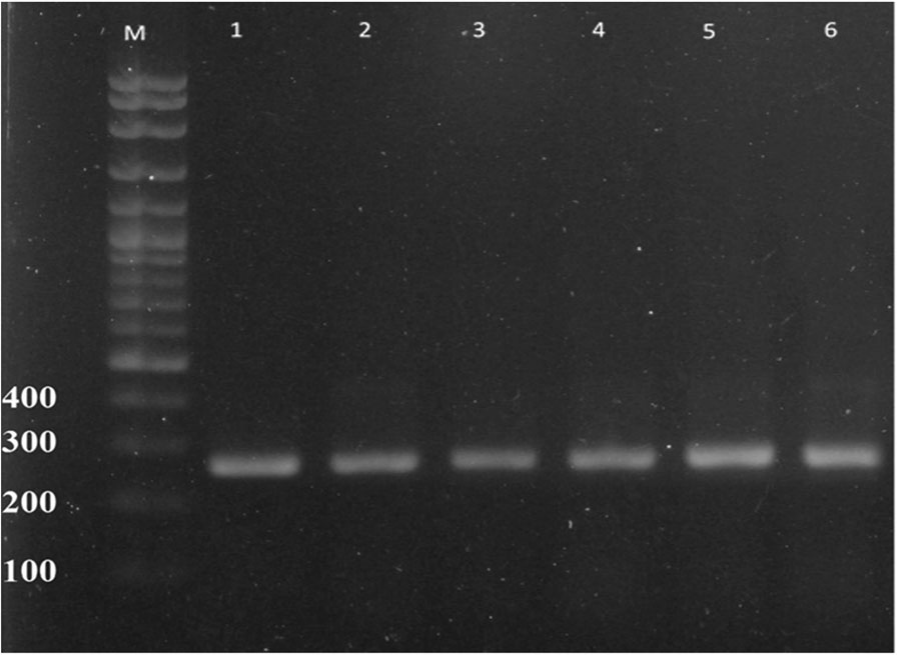
Amplified PCR products of *nuc* gene at (270 bp). Lane M: 100 bp ladder, Lane 1 to 6 positive to *Staphylococcus aureus*

Out of the 200 examined meat samples and 20 hand swabs from human, *S. aureus* was isolated from 29/200 (14.5%) and 11/20 (55%) samples, respectively (Table 2). Isolates were identified as *S. aureus* by positivity in the mannitol fermentation test, catalase test, coagulase (tube) test, acetoin formation test and DNase test. Moreover, these isolates showed positive results using both the Staphytect Plus kit and *nuc* gene detection. (Fig. 1).

**Table 2:** Prevalence of *S. aureus* among the samples from camel meat and hands of the workers

Source and type of the sample	Abattoir (1)		Abattoir (2)		Abattoir (3)		Total samples examined	*S. aureus* positive no. (%)
	Sample no.	*S. aureus* positive no. (%)	Sample no.	*S. aureus* positive no. (%)	Sample no.	*S. aureus* positive no. (%)		
Camel meat samples	62	8 (12.9%)	70	10 (14.3%)	68	11 (16.2%)	200	29 (14.5%)
Human hand swabs	6	3 (50%)	7	4 (57.1%)	7	4 (57.1%)	20	11 (55%)

The detection rates of *S. aureus* in the different abattoirs did not differ significantly (*P* = 0.868 for camel meat and 1.000 for human hand swabs). However, the detection rates of *S. aureus* in camel meat samples and human hand swabs showed that *S. aureus* occurred more frequently in the samples from human (55% vs. 14.5%; *P* < 0.001).

The most common resistance pattern was CHL-FOX-OXA-CLI- SXT-ERY-NV for the camel isolates (*P* = 0.000) and ERY-FOX- OXA-VAN-OFX-SXT for the isolates from human (*P* = 0.426) (Table 3). All isolates that showed resistance to VAN were also resistant to FOX and OXA.

**Table 3:** Frequencies of resistance of *S. aureus* isolates from camel meat and from the hands of workers to singular antibiotics

*S. aureus* isolates	CHL^a^	CLI ^a^	ERY^a^	NV^a^	OFX^a^	FOX^a^	OXA^a^	SXT^a^	VAN^a^
Camel (*n* = 29)	26 (89.7)*	20 (69.0)	17 (58.6)	17 (58.6)*	2 (6.9)	25 (86.2)	25 (86.2)	19 (65.5)	8 (27.6)
Human (*n* = 11)	4 (36.4)	5 (45.5)	7 (63.6)	2 (18.2)	6 (54.5)*	7 (63.6)	7 (63.6)	5 (45.5)	6 (54.5)
Total (*n* = 40)	30 (75)	25 (62.5)	24 (60)	19 (47.5)	8 (20)	32 (80)	28 (70)	24 (60)	14 (35)

*Abbreviations*: *CHL* chloramphenicol, *CLI* clindamycin, *ERY* erythromycin, *NV* novobiocin, *OFX* ofloxacin, *FOX* cefoxitin, *OXA* oxacillin, *SXT* trimethoprim-sulfamethoxazole, *VAN* vancomycin

^*^Antimicrobial resistance of *S. aureus* isolates towards CHL, NV and OFX showed a significant dependence on the host (*P* = 0.001, 0.022 and 0.001, respectively)

^a^Data presented as No. (%)

**Fig. 2:**
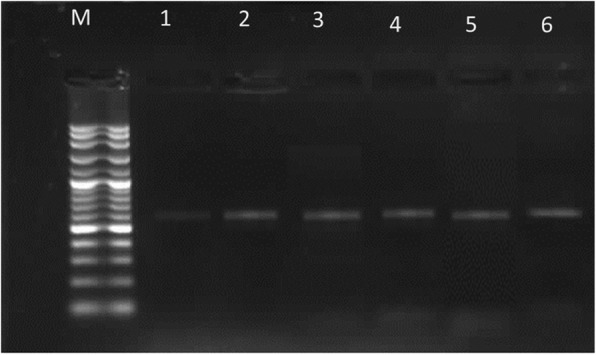
Amplified PCR products of *mecA* gene at (583 bp). Lane M: 100 bp ladder, Lane 1 to 6. Positive to *mecA* gene; results for 6 among the 25 isolates

The *mecA* gene was amplified from all phenotypically FOX-, OXA- and VAN-resistant isolates (Fig. 2).

Of the 40 *S. aureus* isolates examined, 14 isolates (35%) were resistant to VAN, with a MIC> 16 μg/ml. Based on the MIC results, VRSA was detected in 27.6% (8/29) of camel meat samples and 54.5% (6/11) of human hand swabs, without a significant relationship (*P* = 0.111). (Table 4).

**Table 4:** The MIC results of VAN resistance in *S. aureus* isolates from dromedary camels and human

Source	No. of examined samples	MIC (μg/ml)								Total resistant isolates
		0.5	1	2	4	8	Resistant			
							16	32	64	
Camel	29	16	4	1	–	–	5	1	2	8 (27.6%)
Human	11	2	2	1	–	–	2	1	3	6 (54.5%)
Total	40	18	6	2	–	–	7	2	5	14 (35%)

**Fig. 3:**
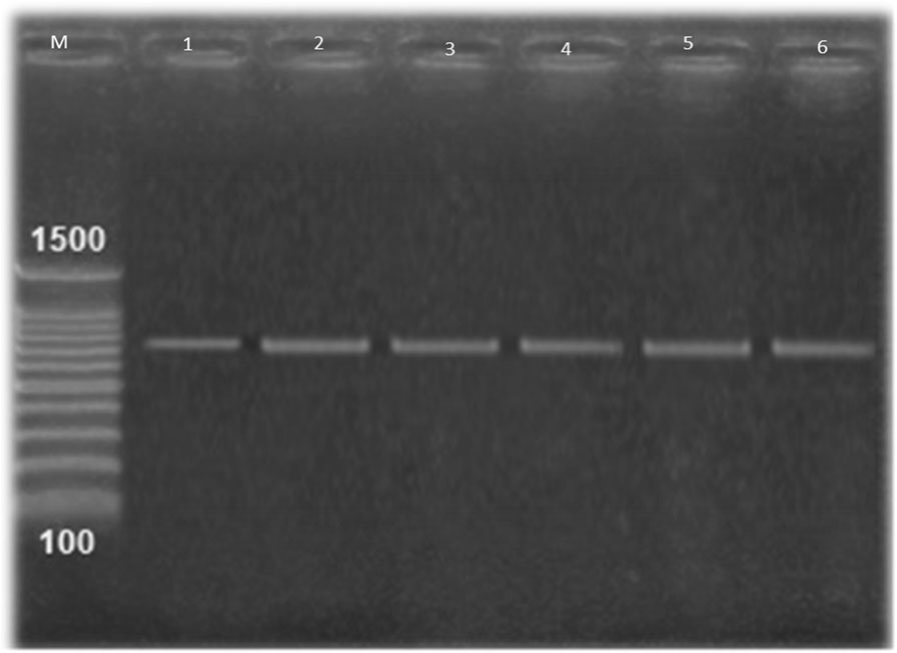
Amplified PCR products of *VanA* gene at (713 bp). Lane M: 100 bp ladder, Lane 1: positive control. Lane 2 to 6. Positive to *vanA* gene of VRSA isolates; results for 5 among the 14 VRSA isolates

**Fig. 4:**
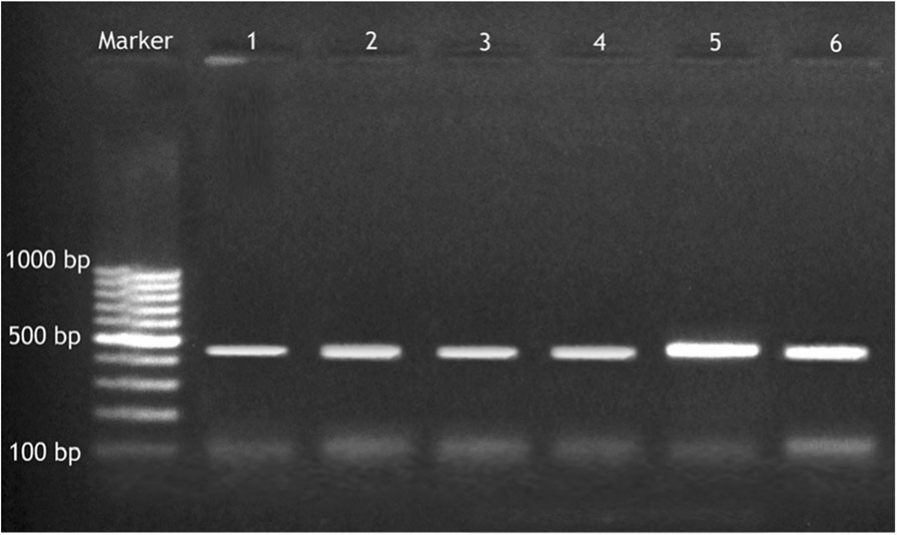
Amplified PCR products of *VanB* gene at (430 bp). Lane M: 100 bp ladder, Lane 1, positive control, lane: 2 to6 positive to *vanB* gene of VRSA isolates; results for 5 among the 14 VRSA isolates

Both the *vanA* and *vanB* genes were amplified from all phenotypically VAN-resistant isolates (14/14,100%) (Figs. 3, 4).

**Fig. 5:**
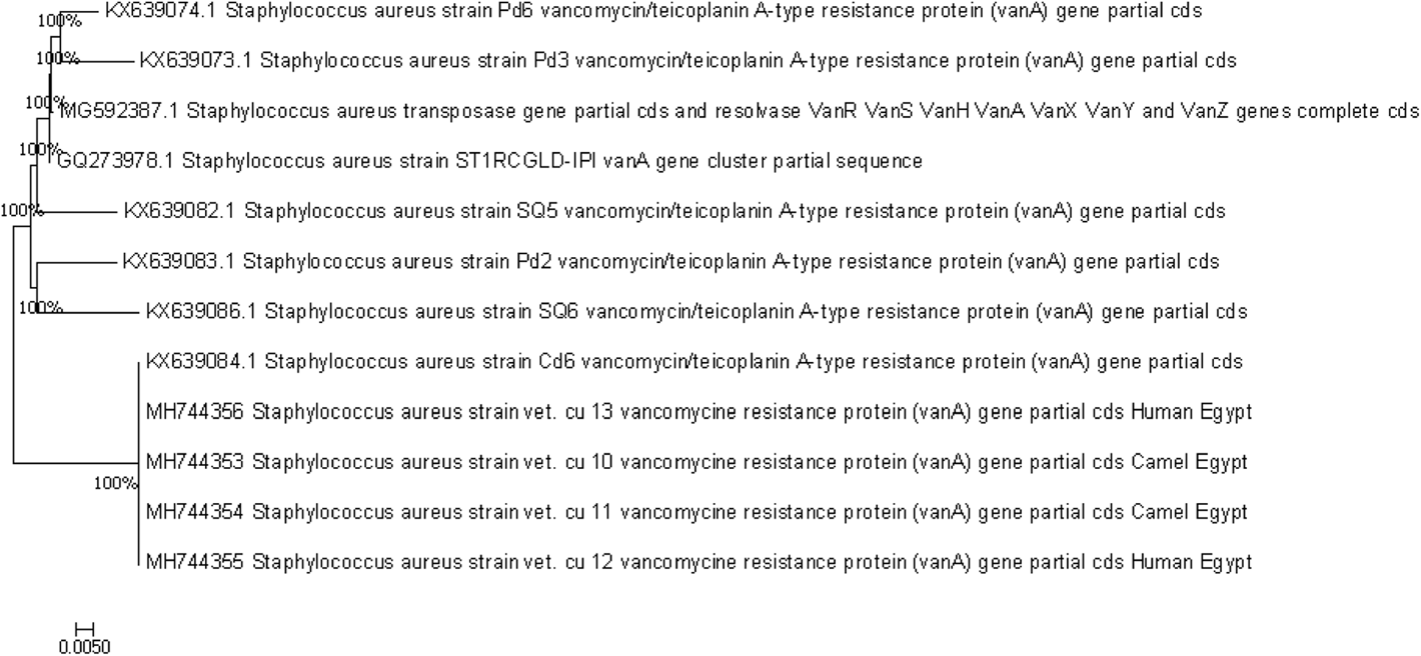
Neighbour joining tree showing the relationship between the nucleotide sequences of the partial coding regions of *VanA* gene of *S. aureus* .The Evolutionary analysis was performed with MEGA version 7

Comparing the sequences of the *vanA* genes revealed 100% homology between the four selected isolates from the camel meat and the hands of the workers in our study and the reference isolate *S. aureus* Cd6 from China, as shown in Fig. 5.

## Discussion

Recently, the epidemiology of *S. aureus* and its newly emerged resistant strains has gained attention in both veterinary and human medicine, particularly because of their zoonotic potential. Although the emergence and spread of resistant *Staphylococcus* strains has been previously reported from apparently healthy pets [13] and pigs [14], there are no definitive data on its prevalence in apparently healthy camels or their role as carriers.

In this study, out of the 200 meat samples from 200 dromedary camels, *S. aureus* was isolated at a high rate (14.5%, 29/200); it was also isolated from 55% (11/20) of the 20 slaughterhouse workers, who were working predominantly at the investigated abattoirs (Table 2).

Very similar *S. aureus* isolation rates (11.7%) were reported in carcass swabs from abattoirs in Addis Ababa, Ethiopia [15]. However, the overall *S. aureus* prevalence in this study was lower than that reported from nasal samples from camels in Nigeria (20.7%) and higher than that reported in human contacts (11.5%) in the same study [16].

Over the past decade, the problem of antimicrobial resistance in the African continent has gained special interest. However, little is known about the real extent of the problem because surveillance for antimicrobial resistance is carried out in only a few countries [17]. In this study, all of the obtained *S. aureus* isolates showed different patterns of multi-resistance to the nine tested antimicrobials. The most common resistance patterns were CHL-FOX-OXA-CLI-SXT-ERY-NV for camel isolates and ERY-FOX-OXA-VAN-OFX-SXT for human isolates (Table 3). The emergence of such resistant strains plays an important role in therapeutic failure in both human and animal infections. The uncontrolled use of antibiotics in human and animals, together with poor diagnostic techniques and inappropriate prescribing by unqualified physicians, exacerbates the problem [18] and constitutes a great challenge for the prevention and control of this pathogen. The same resistance pattern was previously noted in MRSA isolates from an intensive care unit in Hyderabad, southern India, by using the disc diffusion method [16]. Moreover, recently in India, VRSA was identified in 16.7% of MRSA isolates obtained from buffalo nasal and skin samples by using the disc diffusion method [19].

In view of this antibiotic resistance, VAN is now a last-choice antibiotic for the treatment of MRSA, and its use in human and animals is limited [19, 20]. Recently, due to the introduction of other alternative compounds, VAN is no longer an antibiotic of last resort; nevertheless, it is the most frequently used antibiotic in cases of staphylococcal infections [21]. In this study, the isolates showing resistance to VAN were also resistant to FOX and OXA. The *mecA* gene was amplified from all phenotypically FOX-, OXA- and VAN-resistant isolates (Fig. 2). Consequently, there has been concern about the emergence of *S. aureus* strains with decreased susceptibility to VAN.

Although VRSA strains were thought to be rare until recently [22], the present study on the occurrence of VRSA strains in Egypt revealed an increased rate of VRSA isolates. The overall VRSA prevalence was confirmed in 27.6% (8/29) and 54.5% (6/11) of the total dromedary camel and human *S. aureus* isolates, respectively (Tables 3, 4).

Similarly, MRSA was isolated from mastitic femal camels in one study [7] and from camel meat in another study [6]. Moreover, livestock-associated MRSA (LA-MRSA) has been previously detected in the siblings of farmers who were in contact with animals [23], suggesting a potential risk for zoonotic transmission to contacts [24]. In addition, other previous studies showed the acquisition of LA-MRSA from handling meat in Hong Kong [25, 26].

To our knowledge, the prevalence of VRSA has never been investigated among camels in Egypt, which makes it difficult to compare our results with previous data from Egypt.

In this study, we found five VRSA strains with high levels of resistance to VAN (MIC 64 μg/ml): two isolated from camel meat and three isolated from human. The alarmingly high value of these resistant strains and the high prevalence of VRSA strains is of special public health concern (Table 4).

One of the expected mechanisms of VAN resistance in *S. aureus* is the conjugative transfer of plasmids containing Tn1546 and thus the *vanA* gene cluster from VAN-resistant *Enterococcus* spp. (VRE) [11]. Moreover, *vanB* has not been reported for staphylococci thus far.

In this study, we evaluated the presence of *vanA* and *vanB* genes in VAN-resistant *S. aureus* isolates and found that all VAN-resistant isolates harboured both *vanA* and *vanB* genes (Figs. 3, 4). The analysis of the *vanA* gene sequences from isolates from camel meat and human revealed that they were identical to each other, suggesting the zoonotic importance of this pathogen and/or horizontal gene transfer.

In general, VRSA in livestock may come from viscera-contaminated meat during slaughter or from the hands of employees in slaughterhouses, and colonization could pose a potential risk for zoonotic disease transmission [24, 27]. Most of these types of contamination events are of greater concern in Asia and Africa than in Europe, the USA, and Canada [28].

VRSA was isolated from infected or colonized individuals in Turkey and Asiatic countries [29–31]. In Egypt, VRSA strains were not isolated from asymptomatic individuals but were isolated from 4.5% of clinical samples (patients with evident bacterial cutaneous infection) [32]. Clinical infections could result in a major source of community-acquired VRSA in Egypt.

Although the anterior nares are usually the primary site to screen for *S. aureus*, 90% of human nasal carriers also present colonization on their hands [33].

The clear limitation of this study was the lack of nasal swabs from the camels and nasal swabs from the workers; the latter would have been important with respect to VRSA colonization and the risk for further spread among human. Another limitation was the lack of clonal characterization of the VRSA strains isolated from human and animals. Further study based on whole genome sequencing with subsequent core-genome multilocus sequence typing (cg/MLST) is planned in collaboration with an international lab to assess/clarify the zoonotic transmission of *S. aureus* in the camel abattoirs.

## Conclusion

The present study reported the presence of VRSA in camel meat and human in contact with camels in Egypt.

Our research is the first in Egypt to report VRSA in camels, and we urge further comprehensive molecular epidemiological surveillance studies on the extent and potential zoonotic transmission of VRSA in livestock animals. Urgent interventions to control the transmission of these antibiotic-resistant organisms in abattoirs are needed.

## Data Availability

All data generated or analysed during this study are included in this published article.

## Abbreviations

CHL: Chloramphenicol
CLI: Clindamycin
ERY: Erythromycin
FOX: Cefoxitin
NV: Novobiocin
OFX: Ofloxacin
OXA: Oxacillin
SXT: Trimethoprim-sulfamethoxazole
VAN: Vancomycin
